# CHALLENGES AND NOVEL SOLUTIONS TO PROMOTING ADHERENCE TO HOME-BASED COGNITIVE ASSESSMENT

**DOI:** 10.1093/geroni/igad104.0048

**Published:** 2023-12-21

**Authors:** Walter Boot, Ibukun Fowe, Shenghao Zhang, Michael Dieciuc, Andrew Dilanchian

**Affiliations:** Florida State University, Tallahassee, Florida, United States; Florida State University, Tallahassee, Florida, United States; Florida State University, Tallahassee, Florida, United States; Florida State University, Tallahassee, Florida, United States; Florida State University, Tallahassee, Florida, United States

## Abstract

New technologies now allow for home-based self-assessment of cognition to promote the early detection and treatment of cognitive decline. Early detection has a multitude of benefits for the individual and for our scientific understanding of cognitive impairments and possible treatments. However, for these solutions to be effective, they will likely require long-term adherence to assessment protocols to detect longitudinal change, and like many health behaviors, adherence can be a major challenge. This presentation will provide an overview of the Adherence Promotion with Person-centered Technology (APPT) project, which aims to develop a tailored, smart reminder system that infers participants’ contexts and motivations to provide just-in-time support to facilitate cognitive assessment adherence. Among other studies, this overview will include discussion of a recently completed focus group study (N = 42) that was used to understand motivations and potential barriers to home-based assessment and preferences for adherence support, a recently completed pilot study (N = 44) in which the adherence support system was initially tested and refined, and a series of survey studies (total N > 200) that examined older adults’ attitudes toward wearable technologies to detect cognitive decline. The talk will conclude with discussion of an upcoming randomized controlled trial to test the efficacy of the newly developed system with respect to promoting long-term adherence to cognitive assessment via smartphone technologies.

